# Sexual Hormones Determination in Biofluids by In-Vial Polycaprolactone Thin-Film Microextraction Coupled with HPLC-MS/MS

**DOI:** 10.3390/molecules31020255

**Published:** 2026-01-12

**Authors:** Francesca Merlo, Silvia Anselmi, Andrea Speltini, Clàudia Fontàs, Enriqueta Anticó, Antonella Profumo

**Affiliations:** 1 Department of Chemistry, University of Pavia, Via Taramelli 12, 27100 Pavia, Italy; francesca.merlo@unipv.it (F.M.); silvia.anselmi02@universitadipavia.it (S.A.); andrea.speltini@unipv.it (A.S.); 2 Department of Chemistry, University of Girona, C/Maria Aurèlia Capmany 69, 17003 Girona, Spain; claudia.fontas@udg.edu (C.F.); enriqueta.antico@udg.edu (E.A.)

**Keywords:** estrogens, progestins, urine, serum, bioanalysis, green sample preparation, in-vial microextraction, polymeric film

## Abstract

The in-vial microextraction technique is emerging as an alternative sample treatment, as it integrates sorbent preparation, adsorption, and desorption of analytes in a single device before instrumental analysis. In this work, the applicability of polycaprolactone polymeric film, recently used for the in-vial microextraction of sex hormones from environmental waters, is studied in a low-capacity format for unconjugated sex hormones determination in biological samples by HPLC-MS/MS. Its performance was evaluated in urine and serum, achieving extraction in a short time (10 and 30 min, in turn) and satisfactory elution with ethanol, with recovery in the range of 65–111% in urine, 55–122% in bovine serum albumin (BSA) solution, and 66–121% in fetal bovine serum (FBS). In the case of protein matrices, a dilution to 20 g L^−1^ protein content and washing step (3 × 1 mL ultrapure water) afore the elution are required to achieve clean extract, as verified by a Bradford assay. Matrix-matched calibration was used for quantification, obtaining correlation coefficients greater than 0.9929; limits of detection and quantification were in the range of 0.01–0.65 and 0.03–1.96 ng mL^−1^ in urine, 0.02–0.8 and 0.05–2.5 ng mL^−1^ in BSA, and 0.02–1.0 and 0.06–3.0 g mL^−1^ in FBS, respectively. The in-vial polycaprolactone film proved to be reusable for several cycles (up to ten), and the greenness assessment revealed a good adhesion to green sample preparation principles. All these achievements further strengthen its feasibility for efficient extraction/clean-up of trace sex hormones in complex biological samples.

## 1. Introduction

Microextraction techniques are playing a significant role in bioanalysis due to their ability to efficiently extract and preconcentrate target analytes from complex biofluids (e.g., urine, blood, plasma) [1,2,3], but at the same time, they are pursuing the principles of Green Analytical Chemistry (GAC) [4] and Green Sample Preparation (GSP) [5] by cutting the analysis expenses, time, and generated waste.

Among them, thin-film microextraction (TF-ME) stands out due to its higher sorption capacity derived from the higher surface area-to-volume ratio of planar sorptive phases and ease of implementation since the extraction process can be carried out by directly immersing the thin film into the sample [6,7,8]. As an evolution of the TF-ME geometry, in-vial thin-film microextraction has emerged as a more straightforward and compact alternative based on the integration of the sorptive film preparation in the vial where the extraction procedure is performed [9,10,11]. This setup offers high simplicity, coupling sampling by simply adding the sample to the vial, adsorption with analytes partitioning to the coating, direct pouring of the sample for discarding and recovery of analytes by the addition of a low volume of desorption solvent in the same vial. Quite recently, our groups have proposed a microextraction device fabricated to directly form a thin film on the bottom of a glass vial [12]. Polycaprolactone (PCL) is an eco-friendly polymer, well known for its biocompatibility and biodegradability and also with suitable mechanical stability to achieve a stable thin film [13,14]. In our previous work [12], we rationalized the manufacturing steps by dissolving PCL in an environmentally friendly solvent derived from renewable biomass, *viz.* methyl-tetrahydrofuran (Me-THF) [15], improving the sustainability of the film sorptive phase fabrication, thus aligning it with principles of environmental responsibility and GAC/GSP [4,5]. The in-vial PCL films were originally designed for the extraction of sex hormones from large-volume samples such as environmental waters; thus, modifications are required to ensure their applicability to biological samples such as urine and plasma, for which small sample amounts are desirable. The importance of accurate quantification with these hormones in biofluids is linked to the role that these regulators play in human development, fertility, and disease risk and manifestation [16,17,18]. Estrone (E1) and 17-β-estradiol (E2) are endogenous hormones produced mainly by the ovaries; E2 is the predominant circulating female sex steroid hormone in premenopausal women, but it also circulates in small amounts in post-menopausal women, men, and children [19,20]. 17α-ethinyl estradiol (EE2) is a synthetic estrogen, widely used in formulations of contraceptive methods because it is 12 times more effective than E2 when administered orally [16,19]. Progesterone (PROG) plays a role in pregnancy maintenance, and an exogenous form can be administered during early pregnancy to counteract deficiencies [20,21]. On the other hand, synthetic progestins are widely used in human medicine, *viz.* megestrol (MEG) is widely used for long-term contraception, and norgestrel (NORG) is used in second-generation oral contraceptives [12].

The determination of these compounds is predominantly carried out in urine, plasma/serum, and saliva [22]. Monitoring the steroid hormone levels in serum and/or plasma is of great interest and importance in clinical analysis, as they are correlated to endocrine regulation and physiological effects so that altered levels of steroids are indicative of endocrine disorders [18]. However, the direct analysis is not viable due to the huge amount of proteins (ca. 40–80 g L^−1^), which causes serious interference in all steps of the analytical procedure if not removed [17]. On the other hand, protein precipitation has some disadvantages, e.g., incomplete proteins removal and loss of analytes (i.e., steroids) that co-precipitate with plasma proteins, as well as costly and time-consuming procedures. Urinalysis demonstrates higher sensitivity and specificity relative to serum testing, along with practical advantages, *viz.* easier collection compared to plasma, no critical volume restriction, no invasive sampling methods, and interference from circadian secretory variations [22,23]. However, using large volumes of urine for the enrichment of the target analytes could lead to the concentration of potential interferents (e.g., matrix constituents as creatinine, urea, urobilin), which are found in much higher concentrations than estrogens and progestins, thus hampering their extraction and/or resulting in matrix effect in the instrumental measurement [16,24].

Driven by these considerations, exploring new routes to improve the pre-treatment stage in biological analysis of these unconjugated hormones is of actual interest. Therefore, this study is aimed to develop a downsized version of the in-vial PCL films, adapted for processing urine and serum in a miniaturized format, while still ensuring high extraction efficiency, good cleanup, and low analysis times.

## 2. Results

### 2.1. Development of the In-Vial TF-ME Procedure in Urine

**Figure 1:**
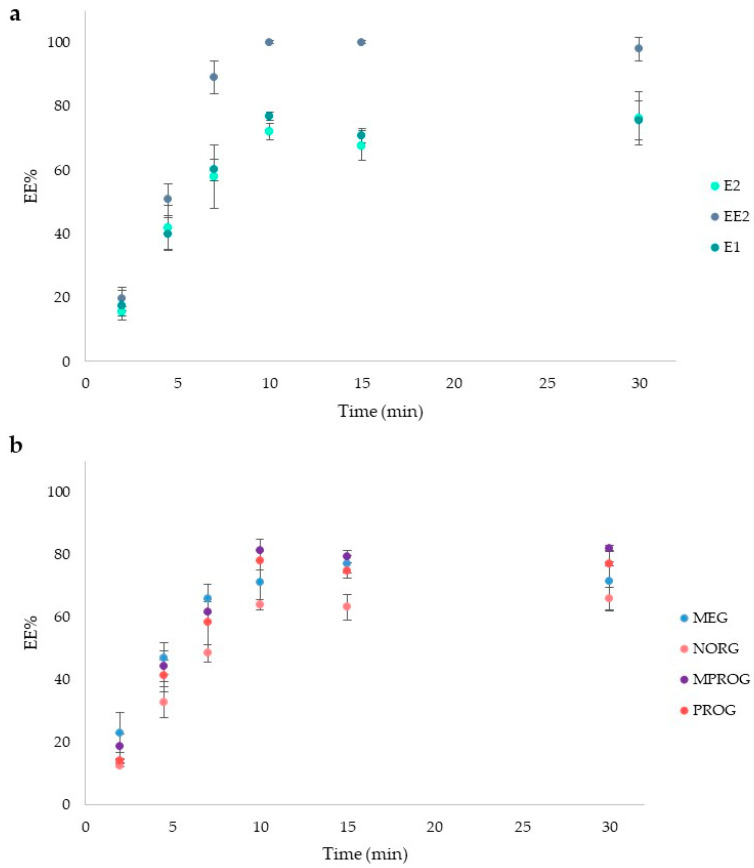
Kinetics experiments. (**a**) Mean extraction efficiency values (EE%, *n* = 3) with standard deviation (Std. Dev.) for estrogens; (**b**) mean extraction efficiency values (EE%, *n* = 3) with standard deviation (Std. Dev.) for progestins.

The in-vial TF-ME procedure was firstly developed in synthetic urine (Section 3.2 for the composition) using the single-factor method [11,25], studying the effects of different parameters on the enrichment performance for target analytes. First, the influence of the extraction time was evaluated within 2–30 min. The results (Figure 1a,b) showed the typical extraction profile with an increase in the EE% (Equation (1) in Section 3.4.1) until the sorption equilibrium was reached, after 10 min. Thus, an extraction time of 10 min was selected as optimum.

**Figure 2:**
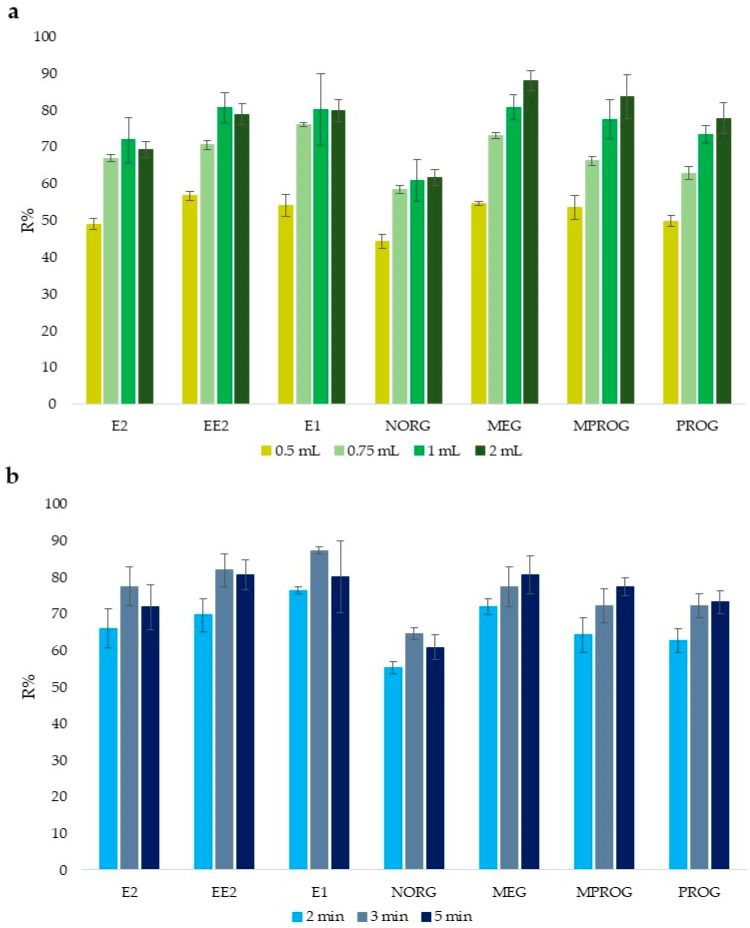
Elution conditions. (**a**) Mean recovery values (R%, *n* = 3) with standard deviation (Std. Dev.), entailing different eluent volume; (**b**) mean recovery values (R%, *n* = 3) with standard deviation (Std. Dev.), entailing different time of agitation.

Regarding the elution step, ethanol (EtOH) was used as the eluent solvent due to its lower toxicity compared to methanol (MeOH) and to its compatibility with the PCL polymer and with the chromatographic system, hence avoiding any solvent change before analysis [12]. The orbital shaker was fixed as the type of agitation because it allowed us to treat 40 samples simultaneously, thus minimizing energy demand and maximizing the sample throughput [12,26]. Elution volume and time were then studied in terms of recovery (Equation (2) in Section 3.4.1). As it can be seen in Figure 2a, elution using 1 mL resulted in the best compromise allowing quantitative recovery and an acceptable enrichment factor (EF) of the steroids in this biological matrix, whereas 3 min was sufficient (Figure 2b) to obtain quantitative elution of the target analytes (R% > 65, RSD% < 7, *n* = 3), further maximizing the sample throughput.

Based on these findings, the in-vial TF-ME procedure was then applied to human urine (Section 3.2 for further details), highlighting a slight decrease in recovery (Figure S1, Supplementary Materials). The influence of the sample volume was therefore investigated at two levels: 2 mL and 4 mL. Based on the results shown in Figure S1, a sample volume of 2 mL was selected, as this provided a higher recovery (R% > 65%, RSD% < 5, *n* = 3), which can be explained by the higher sorbent amount-to-sample volume ratio that turns into a lower interference of the matrix in adsorption [16] and better contact between the sample and the sorptive film.

Finally, in-vial TF-ME was applied to urine samples spiked at lower concentration levels (ng mL^−1^), quantified by HPLC-MS/MS (Section SIV in the Supplementary Materials for the chromatographic conditions). In detail, the accuracy, expressed as R% (Equation (2) in Section 3.4.1), and its associated relative standard deviation (RSD%) were studied in human urine at four different quality controls, namely LLQC (0.5 ng mL^−1^), LQC (2 ng mL^−1^), MQC (20 ng mL^−1^), and HQC (50 ng mL^−1^). As pointed out in Table 1, the recoveries were in the range between 65 and 111%, with RSD below 18%.

Despite the complexity of raw urine, it should be noted that neither sample pre-treatment (e.g., dilution, protein precipitation, centrifugation, filtration) nor washing before the elution is required, as we can still obtain satisfying accuracy and high-quality chromatographic profiles (Figure S4a, Supplementary Materials).

### 2.2. Development of the In-Vial TF-ME Procedure in BSA and FBS

The proposed analytical method for urine was then tested in BSA solution (80 g L^−1^ in PBS, pH 7.2 [17]) as a model plasmatic protein, evaluating the clean-up efficacy by Bradford assay (Section SV in the Supplementary Materials) and recovery. As somehow expected, the residual albumin in the eluate was very remarkable (around 4.8 mg), making impossible the direct analysis by HPLC systems [17]. To abate the residual albumin, after extraction, sequential washings (3 × 1 mL ultrapure water, 1 min each) were performed, affording removal of about 89%, 10%, and 2% of the co-adsorbed protein, respectively, with no loss of analytes. Even if the first washing is already effective in the removal of the protein, the subsequent washings are still necessary to achieve clean extracts to be injected in HPLC-MS/MS. As reported in Table S2 (Supplementary Materials), in these conditions, the protein amount in the eluate was significantly lowered (to about 50 μg), but analytes’ recovery was not quantitative (<25%). A slight increase was noticed, prolonging the adsorption time from 10 min up to 30 min (Table 2) but still remaining not quantitative. Next, the effects of different dilutions (1:1, 1:3; 1:4, 1:5, 1:8 *v*/*v* in PBS) of the BSA solution (80 g L^−1^) were investigated, revealing that recovery is strongly influenced by the dilution (thus by the initial sample protein content), while the residual protein amount was not affected (Table S2, Supplementary Materials). Looking at the results shown in Table 2, a 1:4 *v*/*v* dilution (corresponding to a protein concentration of 20 g L^−1^) was selected, as it strikes a good compromise between recovery and sensitivity while ensuring a satisfactory clean-up. Indeed, the residual protein was around 30 μg, equal to <0.075% compared to the amount in the sample processed (40 mg), corresponding to a residual concentration that did not cause either injection valve clogging or an increase in backpressure in the chromatographic system, in agreement with the literature [17].

On the basis of the above, in-vial TF-ME was carried out at lower concentration levels, achieving satisfactory results, as shown in Table 3. Superimposable results were achieved in charcoal-stripped fetal bovine serum (FBS), herein selected as the blank surrogate matrix, being hormones-free-certified and representative of a serum sample. In terms of clean-up, the protein amount was 37 μg, substantially similar to the results found for BSA solution (Table S2, Supplementary Materials), attesting that the model protein well simulates real samples.

Representative chromatograms from the analysis of the in-vial TF-ME eluates from BSA and FBS are presented in Figure S4b,c (Supplementary Materials), further highlighting the good performance in terms of sensitivity and clean-up.

### 2.3. Analytical Evaluation of the In-Vial TF-ME Followed by the HPLC-MS/MS Method

Once the variables affecting the extraction were established, analytical evaluation was performed according to the figures of merit detailed in Section 3.5 [27]. The analytes are stable in all samples in a wide temperature range (from 30 °C to −20 °C), with differences in terms of trueness lower than 15%. Accuracy was assessed at the ng mL^−1^ level, obtaining satisfactory recoveries (Table 1 and Table 3) and good within-laboratory inter-day precision (RSD < 20%). The injection of blank sample(s) and pure EtOH after QCH samples allowed us to exclude any instrumental carry-over between consecutive analyses. Selectivity was guaranteed by MRM detection, which makes use of the most intense transition precursor/product ions of each compound (Table S1, Supplementary Materials). Actually, no peaks of isobaric compounds or interfering species were present at the steroids’ retention times. The calibration was performed with standards in the matrix, showing a good linearity in the range of 0.5–50 ng mL^−1^ for urine, with R^2^ values higher than 0.9957, and in the range of 2.5–100 ng mL^−1^, with R^2^ values higher than 0.9990 and 0.9929 for BSA and serum, respectively. Matrix effect (ME%) resulted in a consistent signal suppression for progestins (up to 60%) but negligible for estrogens due to the different ionization mode (positive for progestins and negative for estrogens), as previously assessed [17], thus highlighting the importance of the matrix-matched calibration approach. The overall procedure gave method detection and quantification limits (MDLs and MQLs, in turn) suitable for the bioanalysis, as reported in Table 4.

Thus, as a proof of concept, the levels of these sex hormones in the unconjugated form were checked in samples collected from adult volunteers (women, age 25–35) after oral administration of hormonal contraception, as reported in Table S3, Supplementary Materials.

### 2.4. Reusability of the In-Vial PCL Film

**Figure 3:**
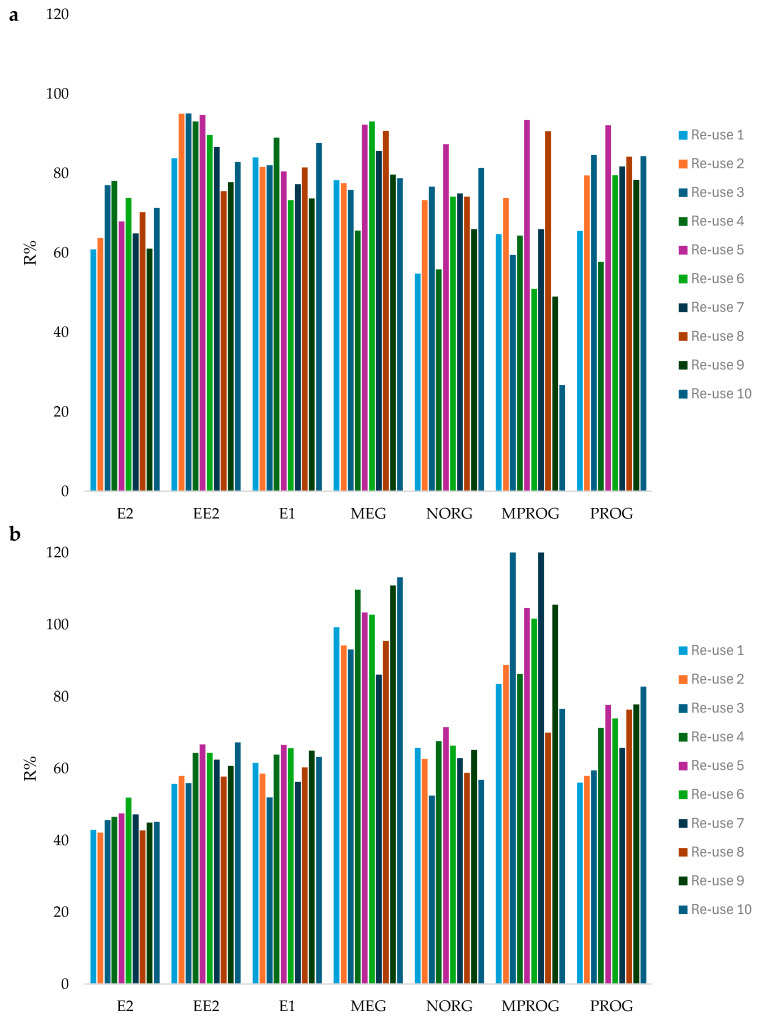
Reusability tests. Mean recovery (*n* = 2) observed for each probe in (**a**) urine samples and (**b**) BSA samples.

In the context of the GSP vision, it is important, and desirable, to have extraction phases that can be reused without a significant loss in efficiency and, at the same time, with no memory effect among biological samples. The reusability of the in-vial PCL film was tested in urine and BSA samples after establishing a cleaning protocol. For this purpose, after the elution, the film was washed with 1 mL of EtOH under an orbital shaker to avoid excessive solvent consumption but, at the same time, to guarantee no carry-over between consecutive extractions. Then, the film was conditioned with 1 mL ultrapure water before the next extraction. Several extractions were performed using the same in-vial PCL film, highlighting a good stability of the sorbent. As shown in Figure 3, the recovery remained constant while the number of extractions increased up to 10, and no carry-over or increased matrix effect was noticed.

### 2.5. Greenness Evaluation

**Figure 4:**
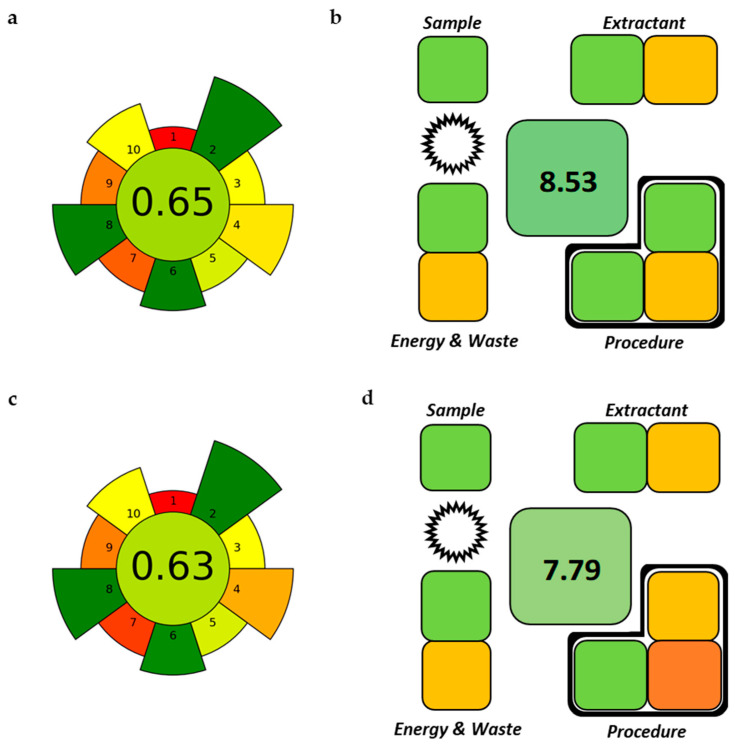
Greenness assessment of the in-vial TF-ME procedure. Graphical output obtained by AGREEprep in (**a**) urine and (**c**) serum, and by SPMS in (**b**) urine and (**d**) serum. For AGREEprep: (1) sample preparation placement; (2) hazardous material; (3) sustainability, renewability, and reusability of materials; (4) waste; (5) size economy of the sample; (6) sample throughput; (7) integration and automation; (8) energy consumption; (9) post-sample preparation configuration for analysis; (10) operator’s safety. For SPMS: sample amount; extractant information (amount and nature of extractant); procedure information (number of steps; extraction time; additional steps after extraction; sample throughput); energy consumption (dispersion; separation; temperature); total waste; reusability of the extractant; The star indicates the reusability and the frame the multiple samples simultaneously processed.

The greenness of the proposed in-vial TF-ME methods was conducted by AGREEprep [28], and by SPMS [29], as these tools are specifically devoted to evaluating the sample preparation in relation with GSP principles. The former enables assessing the greenness through the ten individual GSP criteria (including sampling and instrumental analysis), while the latter takes into account nine parameters divided into four different categories only related to sample treatment stages. The pictograms obtained after the application of each metric, shown in Figure 4, highlighted the adhesion to GSP principles, mainly because the microextraction allows us to process, in a simple way, low sample volume (2 mL), entailing a reusable and biodegradable sorbent in a small amount (ca. 7 mg) and limited energy demand. It is worth noting that the procedure for urine samples received a slightly higher score than the microextraction in serum samples, mainly due to the shorter extraction time (10 min vs. 30 min), number of steps (3 vs. 4), and waste (3 mL vs. 6 mL). Anyway, the sample throughput for both procedures is outstanding (approximately 160 samples *per* hour for urine samples and 60 for serum samples) in comparison with the other literature methods [20,24,26,30,31].

## 3. Materials and Methods

### 3.1. Chemicals and Materials

LC-MS-grade acetonitrile (ACN) and water, ethanol (96% m/m, EtOH), and ammonium fluoride (NH_4_F, for ACS analysis) were purchased from Carlo Erba Reagents (Milan, Italy). For the preparation of the vial extraction device, 4 mL Clear Glass Screw Top Vials supplied by Perkin Elmer (Milan, Italy), polycaprolactone (PCL) provided by Perstorp (Malm, Sweden) and 2-Methyl-tetrahydrofuran (for synthesis, Me-THF) purchased from Merck (Milan, Italy) were used. In this study, the seven steroid hormones, specifically three estrogens (17-β-estradiol, E2; 17-α-ethynylestradiol, EE2; Estrone, E1) and four progestins (Norgestrel, NORG; Megestrol Acetate, MEG, Medroxyprogesterone acetate, M-PROG; Progesterone, PROG) were supplied by Sigma-Aldrich Merck (Milan, Italy). Stock solution of each analyte (1000 mg L^−1^) was prepared in EtOH and stored in the dark (4 °C). Multi-analyte solution (100 mg L^−1^ of each analyte in EtOH) was subjected to step-by-step dilution with EtOH for further use (5 mg L^−1^, 1 mg L^−1^, 10 μg L^−1^). Bradford Reagent (for 0.1–1.4 mg mL^−1^ protein), Bovine Serum Albumin (BSA, >98%), and Charcoal-stripped fetal bovine serum (FBS) were purchased from Sigma-Aldrich (Milan, Italy).

### 3.2. Biofluids

Synthetic urine was prepared according to an adapted protocol [32] and used for the development of the microextraction protocol. This sample was prepared with the following composition: 0.65 g L^−1^ of MgCl_2_·2 H_2_O, 0.65 g L^−1^ of CaCl_2_·2 H_2_O, 2.8 g L^−1^ of KH_2_PO_4_, 1 g L^−1^ of NH_4_Cl, 4.6 g L^−1^ of NaCl, 25 g L^−1^ urea, 1.1 g L^−1^ creatinine, 2.3 g L^−1^ Na_2_SO_4_, 0.65 g L^−1^ Na_3_C_6_H_5_O_7_, and 1.6 g L^−1^ KCl in distilled water. The pH of the solution was then adjusted to 6.5 with a NaOH (0.1 M) aqueous solution and stored in the dark at 4 °C. Urine samples were voluntarily supplied by healthy adult individuals (male and female), allowing us to collect a 500 mL pool of urines over 8 h. The pool (pH 6.4) was then divided into aliquots (with and without spike), immediately frozen, and stored at −20 °C. Before use, aliquots were left to thaw to ambient temperature, and 2 mL sub-samples (either spiked or unspiked) were extracted according to the procedure described in Section 3.4.2. Additional samples, provided by adult female volunteers under different hormonal therapies, were processed in the same way.

Bovine serum albumin (BSA) aqueous solutions were prepared at 80 g L^−1^ in phosphate buffer solution (PBS, pH 7.2) [17] and used to simulate a protein matrix such as human plasma. Charcoal-stripped fetal bovine serum (FBS, 40 g L^−1^ protein amount) was employed as the blank matrix for recovery tests at few ng mL^−1^ concentration levels [17]. Protein samples have been divided into aliquots, which were frozen and stored at −20 °C. Before use, sub-samples were left to thaw at room temperature, vortexed for 20 s at 1400 rpm, and extracted according to the procedure described in Section 3.4.3.

### 3.3. Preparation of In-Vial Polycaprolactone Film

The preparation of the low-volume-capacity vials covered by the PCL film was adapted from our previous work [12] to fabricate a miniaturized version. PCL was dissolved in Me-THF, selected over common toxic organic solvents for its higher environmental friendless [15], for 1 h at room temperature and then at 50 °C for 15 min, under continuous stirring. The in-vial films were prepared by casting the polymeric dispersion (1 mL) into a 4 mL glass vial, followed by a slow evaporation of the solvent in 48 h at ambient temperature. The reproducibility of film preparation was estimated by weighing the vials before and after the film formation, obtaining an average mass of 7.5 ± 5 mg (*n* = 14) for the polymer extraction phase.

### 3.4. In-Vial Thin-Film Microextraction (In-Vial TF-ME)

#### 3.4.1. Development of the Procedure in Synthetic Urine

For the development of the in-vial TF-ME procedure, the sample volume was fixed to 4 mL, corresponding to the maximum vial capacity, whereas the analytes’ concentrations were set to 2 μg mL^−1^. 

The influence of the extraction time was evaluated within 2–30 min in terms of extraction efficiency (EE%), calculated according to the following equation:

(1) $$EE\%=\;(1-\frac{C_{t\;}}{C_{0}})\times 100$$

where C_0_ is the initial concentration (2 μg mL^−1^) and C_t_ is the concentration in the sample after a predetermined contact time (t) obtained by HPLC-UV analysis (Section SIII in the Supplementary Materials for the chromatographic conditions) of the post-contact solutions.

The effects of elution time and volume of eluent on the enrichment performance for target analytes were evaluated in terms of recovery (R%), calculated according to Equation (2):

(2) $$R\%=\frac{amount\;of\;eluted\;compound\;(ng)\;}{iniatial\;amount\;of\;compound\;(ng)\;}\times 100$$

The eluate was injected into the liquid chromatograph for analyte separation and quantification, adapting methods previously developed [12,17]. Two different HPLC systems were used. The development of the in-vial TF-ME procedure in synthetic urine was performed using HPLC-UV, while the method validation was performed with HPLC-MS/MS quantitation for improving sensitivity and selectivity. The description of the chromatographic methods is presented in the Supplementary Materials.

#### 3.4.2. Final Procedure in Urine

**Figure 5:**
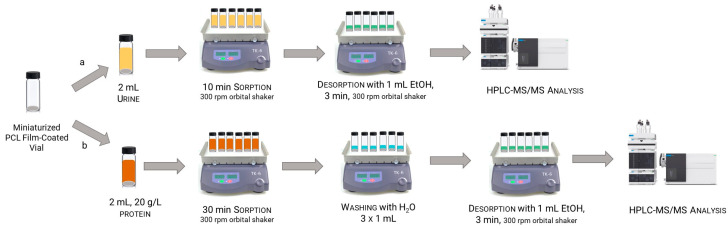
Schematic representation of the analytical workflow for steroids determination in the biological samples: (**a**) urine samples; (**b**) protein matrices.

The urine sample (2 mL, native pH) was placed in the vial and extracted for 10 min under continuous agitation in an orbital shaker (300 rpm), then the sample was easily discarded by simply pouring through the cap. Finally, the analytes were eluted with 1 mL of EtOH under continuous agitation (300 rpm) for 3 min. The use of an orbital shaker for both adsorption and elution allowed us to process 40 samples simultaneously, enhancing the sample throughput. The graphic scheme of in-vial thin-film microextraction followed by HPLC-MS/MS is reported in Figure 5a.

#### 3.4.3. Final Procedure in Protein Matrices

The sample (2 mL, properly diluted with PBS to have a final protein concentration of 20 g L^−1^) was added in the vial and extracted for 30 min under continuous agitation in an orbital shaker (300 rpm). The liquid was removed as above described, and a washing step was performed with 3 × 1 mL ultrapure water (300 rpm, 1 min each). Finally, the elution was carried out with 1 mL of EtOH under continuous agitation (300 rpm) for 3 min, its residual protein content was determined by a Bradford assay (Section SV in the Supplementary Materials), and the eluate was analyzed in HPLC-MS/MS (Section SIV in Supplementary Materials). The graphic scheme of in-vial thin-film microextraction followed by HPLC-MS/MS is reported in Figure 5b.

### 3.5. Analytical Performance of the In-Vial TF-ME Followed by HPLC-MS/MS Method

Analytical evaluation was performed following the ICH guideline [27].

Stability of the target analytes in matrices was evaluated at room and autosampler temperature (20–30 °C), refrigerator temperature (4 °C) and freeze temperature (−20 °C).

Accuracy was assessed in terms of trueness and precision. Trueness was verified by recovery tests in urine, BSA, and FBS and calculated as the ratio of the concentration obtained after the entire procedure on the spiked sample and the concentration expected. The inter-day precision was expressed as RSD % associated to the mean recovery.

Method selectivity was checked by analysis of unspiked samples processed by all steps of the analytical procedure.

To assess linearity, calibration curves were performed in pure solvent (EtOH) and in the in-vial TF-ME eluate from each matrix (matrix-matched calibration curves) in the concentration range of 0.5–50 ng mL^−1^ for urine and 2.5–100 ng mL^−1^ for BSA and FBS. Since in ESI, ionization efficiency of the analytes may be strongly altered by the matrix components, matrix effect (ME%) was calculated for each matrix as:

(3) $$ME\;\left(\%\right)=\left(\frac{b_{m}}{b_{s}}-1\right)\;\times \;100$$

where “b_m_” and “b_s_” are the slopes of the matrix-matched calibration curve and of the calibration line obtained in pure solvent, respectively obtained by ordinary linear lowest squares regression (OLLSR).

MDLs and MQLs (ng mL^−1^) were calculated from the matrix-matched calibration curves as 3.3 and 10 times the ratio between the baseline noise away from the peak tail and the regression line slope, respectively, taking into consideration the whole sample treatment.

## 4. Conclusions

This study provides new insights into the sample preparation (extraction and clean-up) before HPLC-MS/MS determination of unconjugated sex hormones from complex biological samples by reusable PCL film fabricated on the bottom of a small vial. This study clearly combines the benefits of the in-vial microextraction with biological sample protocol, integrating fast and efficient adsorption and desorption towards the target compounds in urine and serum in a single vial prior to LC-MS/MS analysis.

Based on its simplicity, reusability, and controllability, the in-vial polycaprolactone film shows potential for further design as an automated or semi-automated sample processing device to facilitate high-throughput bioanalysis.

## Figures and Tables

**Table 1:** Mean recovery (R%) with RSD% (in brackets) obtained in human urine (2 mL) by the final in-vial TF-ME followed by the HPLC-MS/MS method (*n* = 4).

Analytes	LLQC	LQC	MQC	HQC
E2	84 (14)	66 (7)	79 (9)	86 (5)
EE2	100 (7)	94 (14)	95 (6)	97 (4)
E1	89 (17)	111 (7)	81 (7)	90 (1)
MEG	66 (17)	65 (17)	93 (10)	105 (11)
NORG	100 (17)	83 (6)	80 (11)	88 (1)
MPROG	100 (18)	89 (14)	109 (14)	96 (16)
PROG	80 (12)	71 (6)	89 (6)	102 (12)

**Table 2:** Mean recovery (R%) with RSD% (in brackets) obtained in BSA solution (80 g L^−1^, enriched at 200 ng mL^−1^ of each analyte) at different dilution factors by in-vial TF-ME followed by the HPLC-MS/MS method (*n* = 3, except *n* = 4 for 20 g L^−1^).

Analytes	80 g L^−1^	40 g L^−1 (a)^	27 g L^−1 (b)^	20 g L^−1 (c)^	16 g L^−1 (d)^	10 g L^−1 (e)^
E2	9 (16)	16 (14)	37 (16)	55 (6)	63 (2)	56 (17)
EE2	20 (22)	28 (14)	56 (3)	77 (10)	79 (10)	78 (10)
E1	11 (21)	21 (18)	55 (8)	71 (9)	73 (1)	71 (11)
MEG	37 (17)	48 (18)	108 (13)	122 (8)	123 (13)	97 (4)
NORG	16 (18)	24 (17)	53 (7)	65 (3)	72 (4)	78 (5)
MPROG	37 (17)	46 (17)	84 (15)	85 (17)	70 (17)	83 (22)
PROG	25 (15)	36 (29)	43 (8)	57 (3)	63 (1)	58 (9)

(a) dilution 1:1 *v*/*v* both for BSA and analytes (100 ng mL^−1^); (b) dilution 1:3 *v*/*v* both for BSA and analytes (67 ng mL^−1^); (c) dilution 1:4 *v*/*v* both for BSA and analytes (50 ng mL^−1^); (d) dilution 1:5 *v*/*v* both for BSA and analytes (40 ng mL^−1^); (e) dilution 1:10 *v*/*v* both for BSA and analytes (20 ng mL^−1^).

**Table 3:** Mean recovery (R%) with RSD% (in brackets) obtained in BSA and FBS solutions (2 mL, 20 g L^−1^ protein amount) by in-vial TF-ME followed by the HPLC-MS/MS method (*n* = 3, except *n* = 4 for 200 ng mL^−1^).

Analytes	200 ng mL^−1^		50 ng mL^−1^		10 ng mL^−1^	
	BSA	FBS	BSA	FBS	BSA	FBS
E2	55 (6)	66 (8)	56 (8)	86 (2)	56 (2)	63 (8)
EE2	77 (10)	91 (22)	79 (7)	107 (1)	68 (9)	86 (1)
E1	71 (9)	82 (19)	70 (3)	97 (1)	74 (8)	95 (7)
MEG	122 (8)	88 (20)	102 (7)	121 (1)	102 (13)	114 (18)
NORG	65 (3)	82 (20)	72 (7)	87 (4)	76 (5)	62 (4)
MPROG	85 (17)	90 (15)	98 (18)	95 (10)	97 (18)	92 (3)
PROG	57 (3)	88 (17)	73 (6)	96 (1)	76 (2)	67 (6)

**Table 4:** Method detection and quantification limits (MDL and MQL, in turn) in urine and serum.

Analytes	Urine		BSA		FBS	
	MDL (ng mL^−1^)	MQL (ng mL^−1^)	MDL (ng mL^−1^)	MQL (ng mL^−1^)	MDL (ng mL^−1^)	MQL (ng mL^−1^)
E2	0.02	0.05	0.04	0.1	0.04	0.12
EE2	0.04	0.12	0.05	0.2	0.06	0.2
E1	0.01	0.03	0.04	0.1	0.04	0.01
MEG	0.65	1.96	0.8	2.5	1.0	3.0
NORG	0.14	0.42	0.05	0.2	0.2	0.6
MPROG	0.08	0.23	0.02	0.05	0.1	0.3
PROG	0.02	0.07	0.03	0.1	0.02	0.06

## Data Availability

The data presented in this study are available on request from the corresponding author.

## Supplementary Materials

The following supporting information can be downloaded at: https://www.mdpi.com/article/10.3390/molecules31020255/s1. Section SI: Target Analytes. Section SII: Development of the in vial TF-ME procedure in urine. Figure S1. Mean recovery (R%) with standard deviation obtained in urine samples by the final in-vial TF-ME followed by HPLC-MS/MS method (*n* = 3, except *n* = 4 for synthetic urine). Section SIII. HPLC-UV analysis. Figure S2. Chromatogram of a standard solution prepared in EtOH (3 μg mL^−1^ for each analyte), at λ = 225 nm (red line) and λ = 242 nm (black line). Section SIV. HPLC-MS/MS analysis. Table S1. Tandem mass spectrometry parameters for the target steroid hormones. Figure S3. A TIC chromatogram (black one) overlapped to MRM chromatogram (coloured one) of a standard solution prepared in EtOH (25 ng mL^−1^ for each analyte). Figure S4. A TIC chromatogram (black one) overlapped to MRM chromatogram (coloured one) of in-vial TF-ME eluate from (a) urine sample enriched with 50 ng mL^−1^ for each analyte; (b) BSA and (c) FBS both spiked at 50 ng mL^−1^ for each analyte. Section SV. Bradford Test. Figure S5. Mean calibration curve (*n* = 2) for the Bradford assay (0–40 μg mL^−1^ protein, starting from a 50 μg mL^−1^ BSA standard solution in 0.01 M phosphate buffer, pH 7.2); spectrophotometric determination at λ max 595 nm, 1 cm-optical path cuvette. Table S2. Amount of protein (μg) was measured by Bradford assay (*n* = 2). Section SVI. Analytical performance of the in-vial TF-ME followed by HPLC-MS/MS method. Table S3. Concentrations (ng mL^−1^) of the unconjugated sex hormones in the adult women urine samples analyzed.
