# Supplementary material for: Sexual Hormones Determination in Biofluids by In-Vial Polycaprolactone Thin-Film Microextraction Coupled with HPLC-MS/MS
Source: Molecules. 2026 Jan 12;31(2):255. doi: 10.3390/molecules31020255 (PMC12843708; doi:10.3390/molecules31020255)
Supplement: Supplementary file 1 [file molecules-31-00255-s001.zip › molecules-4075853-supplementary.pdf]

Supplementary Material

Sexual Hormones Determination in Biofluids by In-Vial Polycaprolactone Thin-Film Microextraction Coupled with HPLC-MS/MS

Francesca Merlo [1], Silvia Anselmi [1], Andrea Speltini [1], Clàudia Fontàs [2], Enriqueta Anticó[2] and Antonella Profumo [1,*]

[1] Department of Chemistry, University of Pavia, Via Taramelli 12, 27100 Pavia, Italy; francesca.merlo@unipv.it (F.M.); silvia.anselmi02@universitadipavia.it (S.A.); andrea.speltini@unipv.it (A.S.)

[2] Department of Chemistry, University of Girona, C/Maria Aurèlia Capmany 69, 17003 Girona, Spain; claudia.fontas@udg.edu (C.F.); enriqueta.antico@udg.edu (E.A.)

* Correspondence: antonella.profumo@unipv.it; Tel.: +39-0382-987581

SI. Target Analytes

The target analytes are:

- three oestrogens: 17-β-estradiol, E2; 17- α-ethynylestradiol, EE2; Estrone, E1
- four progestins: Norgestrel, NORG; Megestrol Acetate, MEG, Medroxyprogesterone acetate, M-PROG; Progesterone, PROG

SII. Development of the in-vial TF-ME procedure in urine

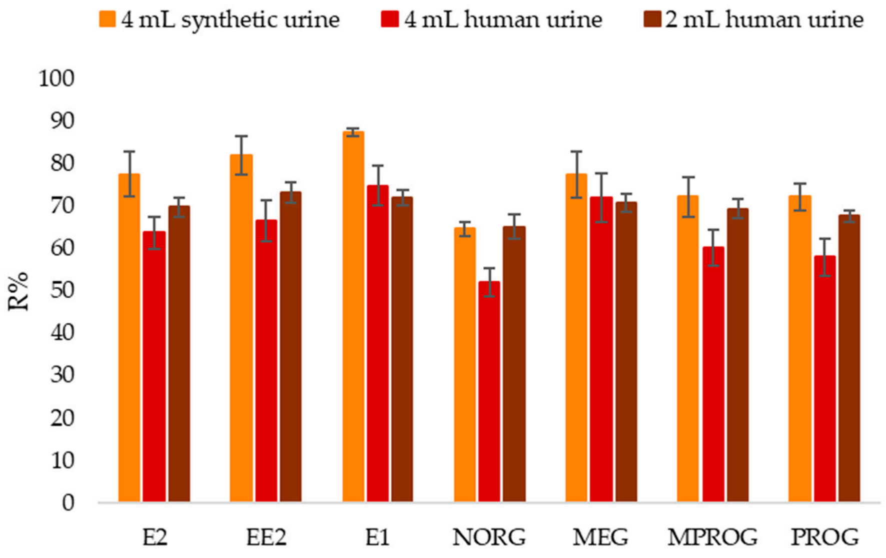

Figure S1. Mean recovery (R%) with standard deviation obtained in urine samples by the final in-vial TF-ME followed by HPLC-MS/MS method (n = 3, except n= 4 for synthetic urine).

SIII. HPLC-UV analysis

High-performance liquid chromatography with ultraviolet detection (HPLC-UV) was employed for the development of method in synthetic urine. A LC-20AT solvent delivery module equipped with a DGU-20A3 degasser and interfaced with a SPD-20A detector from Shimadzu (Milan, Italy) was employed. The analytes were separated with a Supelco Ascentis® C18 (250 × 4.6 mm, 5 μm) column coupled with a similar (2 × 2.1 mm, 5 μm) guard-column. The mobile phase was (A) water, (B) ACN, according to the following elution program: 30% B isocratic for 4 min, linear gradient from 30 to 90% B until 16 min, then to 95% B until 19 min, finally to 98% B until 20 min (kept for 5 min). The flow rate was fixed to 1 mL min^{-1} and the sample volume injected manually was 20 μL. The detection wavelengths were 225 nm for estrogens and 242 nm for progestins. Calibration standard solutions (0.25–25 μg mL^{-1}) were prepared in EtOH. A typical chromatogram is reported in Figure S2.

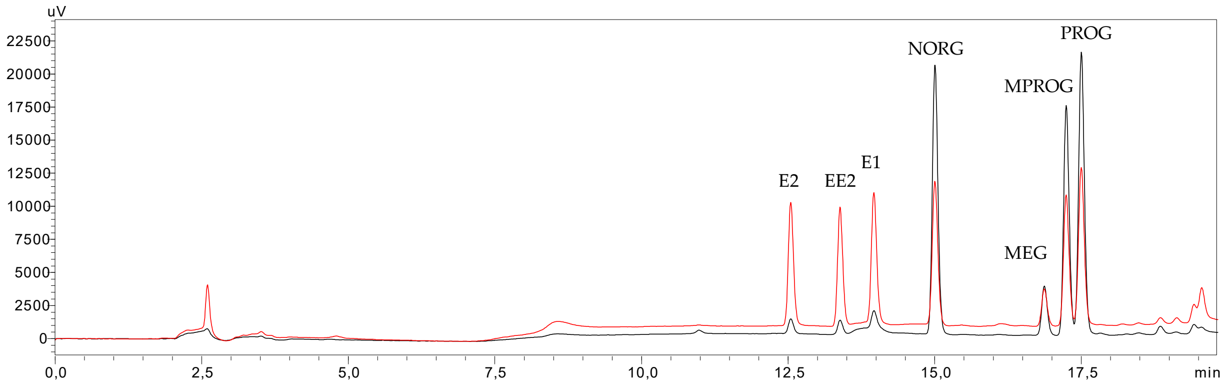

Figure S2. Chromatogram of a standard solution prepared in EtOH (3 μg mL^{-1} for each analyte), at λ= 225 nm (red line) and λ=242 nm (black line).

SIV. HPLC-MS/MS analysis

High-performance liquid chromatography coupled to tandem mass spectrometry (HPLC-MS/MS) was used for the method validation. Mass spectrometry analyses were carried out on Agilent 1260 Infinity coupled with an Agilent 6460C MS spectrometer ESI-MS/MS system (Cernusco sul Naviglio, Italy). ZORBAX Eclipse Plus C18 Rapid Resolution (100 mm × 4.6 mm, 3.5 μm) column preceded by a pre-column Supelco Supelguard Ascentis C18 (2 cm × 2.1 mm, 5 μm) was used for the chromatographic separation at 25 °C (± 0.8 °C). The sample injection volume was 10 μL, directly injected by the HPLC autosampler. The mobile phase consisted of (A) aqueous 1 mM NH_4F in H_2O and (B) ACN, and the elution was performed in a gradient mode according to the following program: linear gradient from 30% to 85% B in 3 min, maintained for 8 min, and to 98% B in 0.5 min, maintained for 1 min, followed by a re-equilibration time of 7 min. The MS operating parameters optimized by Agilent Mass Hunter Source Optimizer Software (Agilent, Santa Clara, CA, USA) were as follows: drying gas (N_2) temperature 350 °C; drying gas flow 12 L min^{-1} ; nebulizer 50 psi; sheath gas temperature 400 °C; sheath gas flow 12 L min^{-1}; capillary voltage 4000 V positive and 3000 V negative; nozzle voltage 0 V positive and 1500 V negative; electron multiplier voltage (EMV) 200 V positive and 0 V negative; cell accelerated voltage (CAV) 4 V positive and 1 V negative. All analytes were detected in multiple-reaction

monitoring (MRM) mode, by setting a polarity-switching tool and using the most intense transitions of each compound as reported below.

Table S1. Tandem mass spectrometry parameters for the target steroid hormones.

Analyte	Precursor Ion (m/z)*	Product Ions (m/z)	Dwell Time (ms)	Fragmentor Energy (V)	Collision Energy (V)	Polarity
E2	271	183.3<br>143.3	100	166	50<br>64	Negative
EE2	295	145.1<br>143.1	100	154	44<br>68	Negative
E1	269	145.1<br>143.1	75	148	55<br>60	Negative
MEG	385.4	120.9<br>109	50	100	20<br>25	Positive
NORG	313	245<br>109<br>91	30	80	20<br>25<br>30	Positive
MPROG	387	327.4<br>123.1	75	106	8<br>24	Positive
PROG	315	109<br>97	50	94	24<br>20	Positive

[1] $[M+H]^+$ for positive ionization, $[M-H]^-$ for negative ionization

A TIC chromatogram (black one) overlapped to MRM chromatogram (coloured one) of a standard prepared in EtOH (25 ng mL^{-1}) is reported in Figure S3.

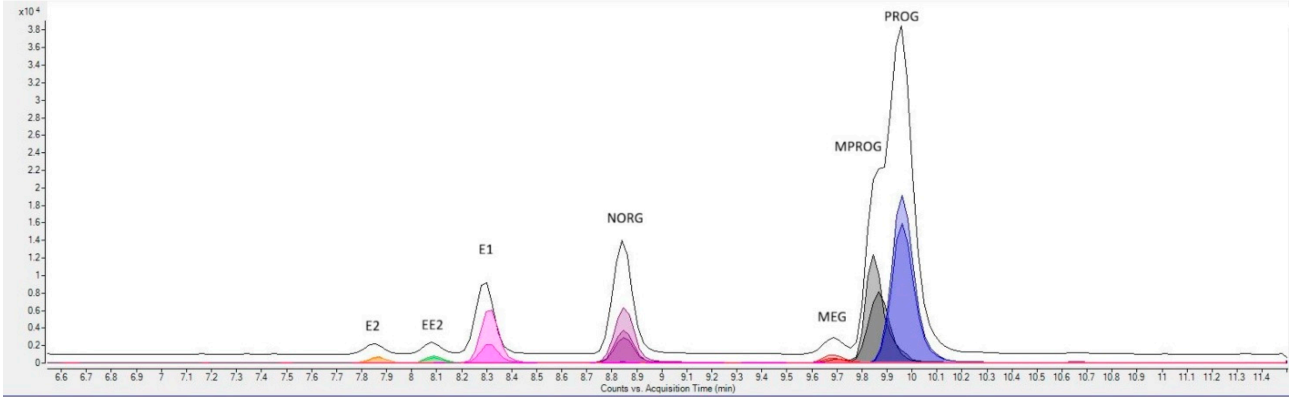

Figure S3. A TIC chromatogram (black one) overlapped to MRM chromatogram (coloured one) of a standard solution prepared in EtOH (25 ng mL^{-1} for each analyte).

The chromatogram of in-vial TF-ME eluate from (a) urine, (b) BSA and (c) FBS is reported below.

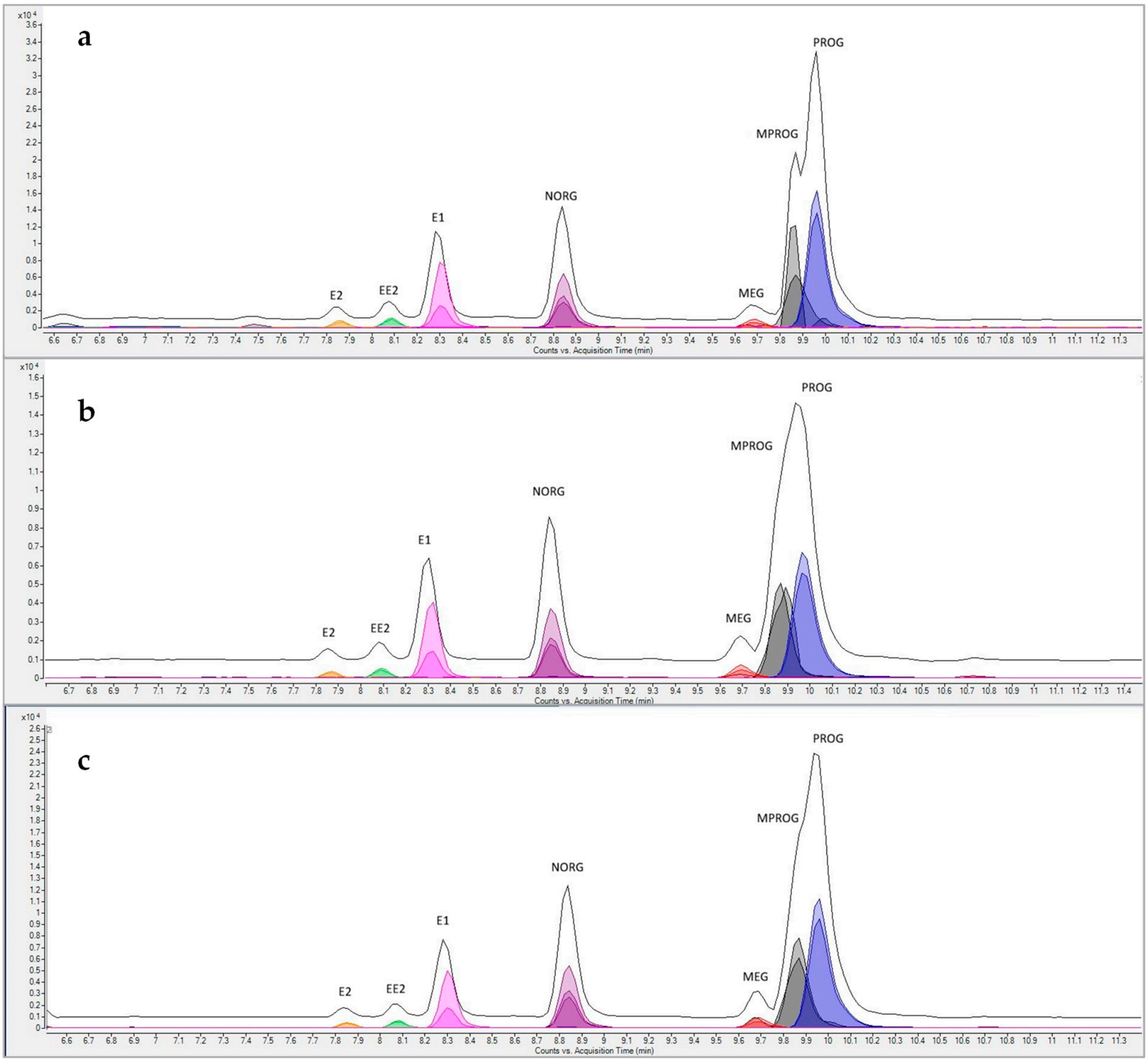

Figure S4. A TIC chromatogram (black one) overlapped to MRM chromatogram (coloured one) of in-vial TF-ME eluate from (a) urine sample enriched with 50 ng mL^{-1} for each analyte; (b) BSA and (c) FBS both spiked at 50 ng mL^{-1} for each analyte.

SV. Bradford Test

Bradford assay is a colorimetric protein assay, based on an absorbance shift of the dye Coomassie Brilliant Blue G-250. The binding of the protein stabilizes the unprotonated form of the Coomassie dye, causing a shift in the Amax to 595 nm. The change from red to blue upon binding protein is measured spectroscopically. Since the amount of the blue anionic form is proportional to the amount of protein in the sample, the amount of protein in a sample can be estimated by measuring the absorption at 595 nm.

Firstly, a calibration curve was constructed (0–40 μg mL^{-1}) starting from a Bovine Serum Albumin solution (BSA, 50 μg mL^{-1} in phosphate buffer solution, pH=7.2), as reported in Figure S2.

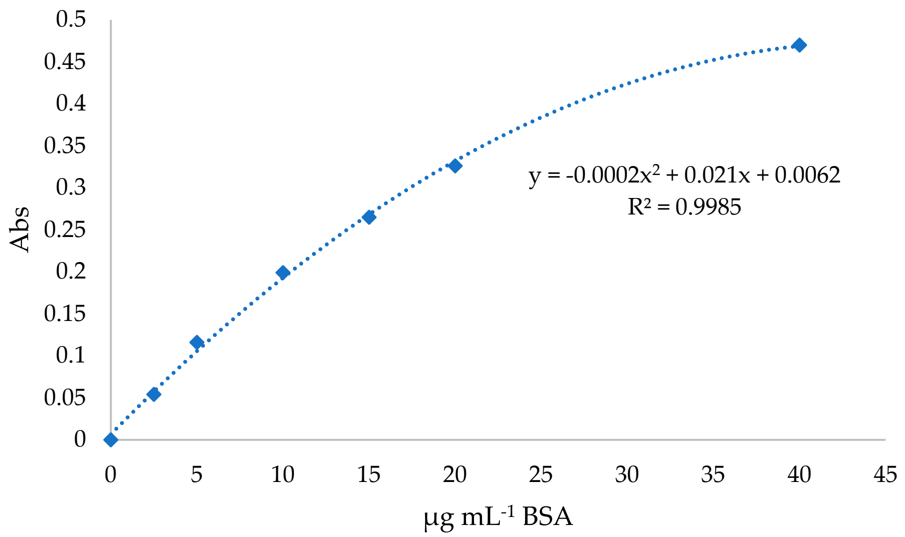

Figure S5. Mean calibration curve (*n*=2) for the Bradford assay (0-40 μg mL^{-1} protein, starting from a 50 μg mL^{-1} BSA standard solution in 0.01 M phosphate buffer, pH 7.2); spectrophotometric determination at λ max 595 nm, 1 cm-optical path cuvette.

Then, the amount of protein was measured in the different sample, achieving the results gathered in Table S2.

Table S2. Amount of protein (μg) was measured by Bradford assay (*n* = 2)

BSA	μg BSA in eluate
80 g L^{-1}	50
40 g L^{-1} (dil 1:1)	45
27 g L^{-1} (dil 1:3)	44
20 g L^{-1} (dil 1:4)	37
16 g L^{-1} (dil 1:5)	38
10 g L^{-1} (dil 1:8)	35
FBS (dil 1:4)	37

SVI. Analytical performance of the in-vial TF-ME followed by HPLC-MS/MS method

Table S3. Concentrations (ng mL^{-1}) of the unconjugated sex hormones in the adult women urine samples analyzed.

Analytes	Female 1	Female 2
E2	< MDL (< 0.02)	0.6
EE2	< MDL (< 0.04)	0.7
E1	0.5	0.6
MEG	< MDL (< 0.65)	< MDL (< 0.65)
NORG	< MQL (< 0.42)	0.5
MPROG	< MQL (< 0.23)	0.6
PROG	0.4	0.3
